# Editorial

**Published:** 1904-08-15

**Authors:** 


### EDITORIAL.

A THREE YEAR-COURSE AGAIN.—As we predicted in our last issue, the called meeting of the National Association of Faculties decided to return to a three-years' course of instruction. The term is to be eight months, and the entrance requirements are to be left as before. This action will probably be accepted as final by all schools, and establishes this matter for a long period so far as this organization is concerned. It was evident at the close of the Washington meeting that the matter had not been satisfactorily settled, but it was hoped that the decision there reached might influence those schools favoring the three-year term to accept that decision. There was no doubt but that the profession would approve the four-year course, and had all the schools adopted it in good faith last year, the whole question would have taken care of itself in a few years. The addition of one month to the teaching time is a slight compensation, but it will not meet the approval of some schools. An increase in the entrance repuirements would be a much more substantial gain. And it is in this direction that we have hopes for advances in the future, for it is hardly probable that the association will agree to extend the time further in another decade.

—✦—

THE STATE EXAMINING BOARDS AND THE THREE-YEAR COURSE.—What will be the attitude of the examiners in those States where a four-year course of instruction is required for registration? This is a question that is now of interest to schools located in these States, and it will, no doubt, cause them considerable anxiety to reconcile matters until such time as the State laws can be changed, if such an action is possible. Now that the Faculties Association has settled its standard of graduation, it devolves upon the examiners to decide whether this standard is acceptable. They may accept this standard as qualifying for "reputability," but will they deem it sufficient for registration? It is not improbable that the examiners will now interest themselves in determining what shall be a suitable curriculum to qualify for registration, and make it a substitute for examination. This is a suitable work for this organization to do. It could set up a standard of qualification for registration that would be accepted in all States and which would permit a transfer of residence on certificate. Such an action would meet with the universal approval of the profession, and it would have the immediate effect of bringing the colleges up to it, whether it seemed right and feasible to them or not.

—✦—

Financing the International Congress.—Dr. C. S. Butler, of Buffalo, who is Chairman of the Finance Committee, made a statement before the recent meeting of the Michigan Dental Association as to the financial affairs of the congress. He said that the congress, while having an international character, because of its origin and authority, was being held in this country on our invitation, making us responsible as hosts for its proper conduct. The congress is now fully organized and the program is practically completed. In papers and clinics it will probably be the most notable meeting ever held. An effort is being made to curtail the papers to ten in each section, but one section has already seventeen papers offered. Consequently, the printed proceedings will be the most valuable literary product of the year and possibly for a long time. It is estimated that the proceedings will contain nearly 2,000 pages of printed matter. These proceedings are given free to all who contribute ten dollars as a membership fee. It is estimated that they will actually cost eight dollars to print and bind.

so that only two dollars of every membership fee will be available for expenses of the congress. It is estimated that the expenses of the congress will be in the neighborhood of $35,000, and to raise this amount will require a lot of hard work, unless every one, who can, takes out a membership. The resources outside of membership fees are dental societies, National, State, and district, and local dental colleges, dental dealers and manufacturers. New York State societies have given $1,650. Every State society, except one, that has been asked has contributed something, and the members of that society agreed to take out individual memberships. Between forty and fifty manufacturing concerns have contributed. More than one-half of the dental colleges have contributed sums varying from twenty to one hundred dollars each. A contribution has been received from the Dental Society of Honolulu. Twenty-six State societies have so far made contributions. In fact, so much progress has been made that there is no question but that the expenses will be met, if the members of the profession will come forward and take out memberships. There ought to be at least three thousand memberships.

The value of the membership fee is not all included in the copy of the transactions, but the congress will be open only to such as have taken out the membership. The meetings, clinics, exhibits and entertainments will be open only to such as are members of the congress. This rule will be rigidly enforced.

It has been stated that the funds are likely to be extravagantly used up in junketing trips and banquets to the officers and distinguished guests. In refutation, Dr. Butler stated that claims for traveling expenses of committee men and other legitimate expenses of that kind will only be paid after all other expenses have been met. Printing bills, postage accounts, hall rents and such other necessary expenses will be first met before any personal claims will be allowed. In addition a detailed statement of all expenses will be mailed to every member of the congress at the close of the business transactions.

LECTURESHIP VACANT.—The July *Dominion Dental Journal* contains the statement that the position of Lecturer on Physiology is vacant and applications will be received up to August 1st. The specifications are brief, and call only for a "minimum duty of fifty lectures to each session." This is rather a novel way of filling vacancies in college faculties. We are not prepared to say that it may not have advantages over the prevailing system. It will at least secure, without much trouble, a list of eligibles from which the choice may be more easily made, and the "call" can then be issued in conventional form. It seems, however, as though it was taking on competitive business methods, where tradition has been thought to perpetuate other ideals.

INTERNATIONAL DENTAL CONGRESS.—Before another issue of the REGISTER the Fourth International Congress will have passed into history. We are giving considerable space of this issue to an announcement of it, and the program so far as made up at this time. It will undoubtedly be the best dental convention ever held on this continent, and every dentist who can should attend. There will be something of value and interest for every one. It will be worth while to get a view of the men who are leading in thought and invention. It will be many years probably before another like occasion will be available. A membership costs ten dollars, and secures all the benefits including a copy of the transactions, which every man ought to have.

